# Information-Theoretic Quantification of Dedifferentiation in the Aging of Motor and Executive Functions

**DOI:** 10.3389/fnagi.2021.634089

**Published:** 2021-08-20

**Authors:** Erik Chihhung Chang

**Affiliations:** Action and Cognition Laboratory, Institute of Cognitive Neuroscience, National Central University, Taoyuan City, Taiwan

**Keywords:** Fitts' law, majority function, BIT, generalized linear mixed models, dedifferentiation

## Abstract

A central account of cognitive aging is the dedifferentiation among functions due to reduced processing resources. Previous reports contrasting trends of aging across cognitive domains mostly relied on transformed scores of heterogeneous measures. By quantifying the computational load with information entropy in tasks probing motor and executive functions, this study uncovered interaction among age, task, and load as well as associations among the parametric estimates of these factors at the individual level. Specifically, the linear functions between computational load and performance time differed significantly between motor and executive tasks in the young group but not in the elderly group and showed stronger associations for parameters within and between tasks in the elderly group than in the young group. These findings are in line with the dedifferentiation hypothesis of cognitive aging and provide a more principled approach in contrasting trends of cognitive aging across different domains from the information-theoretic perspective.

## Introduction

Aging impacts cognitive functions in a variety of manners: while some functions have been found to deteriorate (e.g., fluid intelligence), others seem to remain relatively stable across years of later adulthood (e.g., crystallized intelligence; Park and Gutchess, 2002; Salthouse, 2019). As the population worldwide rapidly grows older, precisely depicting trajectories of aging for different cognitive functions have great values from various perspectives, including clinical applications, policymaking, life span education, industrial research, and development, etc. At the group level, both cross-sectional and longitudinal reports comparing standardized scores of cognitive functions across life span suggest distinctive progression of aging-related impacts on different cognitive domains (Park and Gutchess, 2002; Salthouse, 2019). Outlining the landscape of aging across multiple cognitive domains is not a trivial business, considering the wild variety of the ways that different cognitive functions within a domain are operationally defined, measured, and compared. This study aims to examine this landscape by exploring the difference and relationship between two cognitive domains that are known to be susceptible to the impacts of aging, namely, the executive, and motor control.

As cognition comprises a vast array of diverse functions and researchers having accumulated numerous paradigms in studying them, it is almost unavoidable to apply a certain scheme of standardization when constructing any panoramic view of cognitive aging. For example, in the most widely applied procedure, *z*-standardization, the observations are demeaned, and divided by the sample SD. In the context of cognitive aging, the standardized performance indices of different abilities as functions of age are then contrasted for assessing the trend. As one loses the absolute scale of the performance measure, the scope of interpretation from the *z*-score approach is limited to the comparison of general trends in the ability of each individual. Further exploration of the relationships among trends in different abilities can be misleading, due to the fact that standardization often distorts the distances between observations and the multivariate distributions of cross-sectional (Fischer and Milfont, 2010) and longitudinal data (Moeller, 2015). While alternative ways of transforming heterogeneous measures have been proposed to overcome the above problems (Little, 2013), a fundamental resolution is to design tasks for different domains in ways that independent variables vary along a commensurable dimension. Specifically, this is viable by applying the information theory (Shannon and Weaver, 1949) in quantifying the computational load involved in various cognitive functions (Fan, 2014).

Although the information theory sits at the core of the “cognitive revolution” (Neisser, 1976), in practice, it served mostly as a conceptual metaphor. Seldom do experiments in cognitive psychology quantitatively relate the amount of information embedded in stimuli to be processed to mental operations. With the appropriate experimental design, the information-theoretic approach offers a simple and clear measurement of cognitive functions. For example, the executive function can be assessed with tasks that involve uncertainty processing, such as the majority function task (MFT; Fan et al., 2008; Wang et al., 2011; Fan, 2014). In this task, participants are shown a number of left/right arrows and asked to indicate the direction in which the majority of the arrows are pointing. By manipulating the set size and congruency (i.e., the ratio of the number of left/right arrows) in each trial and with assumptions of searching strategies (i.e., exhaustive, self-terminating, or grouping), the per-trial computational load can be quantified with the entropy estimate, “bit.” Fan et al. (2008) determined that grouping-search outperformed the other two algorithms in capturing the linear relationship between the reaction time (RT) and computational load in MFT [RT = *a* + *b*∙log_2_(*s*), *s* indicating the average number of arrowheads to be scanned in a trial], where the slope (*b*) indicates how much more time it takes to process per bit of load increase (i.e., processing efficiency). In contrast, the intercept (*a*) represents the processing time at the lowest possible load (0 bit), which is the binary choice RT to a single arrowhead.

While MFT demonstrates how information-theoretic approach can metrically quantify efficiency in the executive function, similar applications of metrical quantification have also been documented in the literature of human motor control. The speed-accuracy trade-off has long been considered a fundamental property of human motor behavior (Woodworth, 1899). Fitts (1954) attributed it to the limited capacity of information transmission in the sensorimotor channel. This central limit forces the duration of performing a task proportional to the amount of information (in bits) required for controlling each targeting movement. The amount of information, coined as the index of difficulty (ID), has the form of the ratio of the target distance to its width. Hence the Fitts' law is expressed as follows: movement time (MT) = *a* + *b*∙ID = *a* + *b*∙log_2_(*A*/*W* + 1) (direct analogy with Shannon's information theorem; MacKenzie, 1992). The slope (*b*) indicates how much additional processing time it requires per bit of ID increase. The meaning of the intercept (*a*) has several interpretations, including unavoidable delay in the psychomotor system (Fitts and Radford, 1966), extra feedback processing time, uncontrollable muscle activity at the beginning or end of the movement task (MacKenzie, 1992), and RT (Fitts and Peterson, 1964). Alternatively, it has also been proposed that Fitts' law is just an approximation of the function of a more general motor circuit model (Beamish et al., 2006), and the intercept in the Fitts' law reflects the consequence of delay processing in the circuit.

The majority function and Fitts' law represent quantitative principles of uncertainty processing in executive and motor functions, respectively. Taking advantages of these two experimental paradigms affording analyses based on the information-theoretic measures can be a way to avoid issues concerning score standardization when carrying out studies comparing performance in different tasks. Regarding the impacts of aging on MFT and Fitts' law, so far no study has documented the difference between the young and elderly groups in MFT performance. However, based on the observations of reduced efficiency in component abilities relevant to MFT, such as visual search, working memory, and conflict resolution, one can reasonably expect elevated intercept and slope of MFT for the elderly group. In contrast, the characteristics of Fitts' law in the later stage of life have been inspected quite thoroughly. It has been quite well-established that, in a variety of Fitts' tasks, the elderly group showed both steeper slopes (Rey-Robert et al., 2012; Temprado et al., 2013) and longer MTs (Welford et al., 1969; York and Biederman, 1990; Teeken et al., 1996; Ketcham et al., 2002). The less efficient processing of the elderly group in both paradigms may or may not have common causes, depending on how well the functional parameters are associated between paradigms, as compared with the young group.

Sleimen-Malkoun et al. (2013) compared Fitts' law and Hick–Hyman's law in young and elderly groups. In contrast to Fitts' law, the Hick–Hyman's law describes the choice reaction time as a linear function of the information entropy of response selection, namely, the binary logarithm of possible Stimulus-Response (S–R) associations, while fixing the complexity of the motor response at the lowest possible level (Hick, 1952; Hyman, 1953; Hawkins et al., 2012). The authors found that while the slopes of the two laws were not statistically separable in the elderly group, the young group showed larger slopes in the Fitts' law than the Hick–Hyman's law. They consider the findings providing evidence for the dedifferentiation view of cognitive aging: owing to the reduction in cognitive resources, distinctive processes at younger age shifted to recruit common resources at an older age, and thus become more similar to each other (Lindenberger and Baltes, 1994; Baltes and Lindenberger, 1997). Although this study demonstrated a novel way of quantitatively contrasting different cognitive domains without transforming the raw data, there are some rooms in the methodological aspects to be further explored, including (1) linear functions were estimated over the group mean RT, which usually overestimates the explained variance; (2) the comparison between tasks qualitatively relied on outcomes of separate ANOVAs because the levels of the difficulty factor in the two tasks were not identical (i.e., not fully “crossed”) and cannot be analytically compared within one single fixed-effect type of model; and (3) trial-by-trial variation within subjects was ignored, which may miss information embedded at the individual level. To deal with these issues, one has to carry out analyses that can take the trial-by-trial variation at the individual level into account and can deal with unbalanced design.

This study will compare aging in the domains of motor and cognitive functions with experimental paradigms manipulating computational load along the same information metric and adopt statistical models that can adequately and quantitatively afford the experimental design. Specifically, the linear functions of the MFT and Fitts' law in the elderly and young groups will be estimated with the generalized linear mixed model (GLMM) to examine the interaction among age, task, and computational load. This will reveal differences between tasks and age groups at the group level (fixed-effect) and at the same time also allows the exploration of correlations between factors, which require estimates of the relationship at the individual level (random-effect). Under the research framework of this study, the dedifferentiation hypothesis would predict not only more significantly different performance–load relationship in the young group than the elderly group but also stronger correlation between individual parameter estimates in the elderly group than the young group.

## Methods

### Participants

Thirty-three elderly and 40 young participants were tested in this study after given informed consent. The elderly participants (mean age: 69.9 years, 95% CI = [67.1; 72.6]; mean education: 11.7 years, 95% CI = [10.5; 12.9]) were community dwellers in the close proximity of National Central University (NCU). The young participants (mean age: 22.7 years, 95% CI = [22.0; 23.3]; mean education duration: 16.3 years, 95% CI = [16.1; 16.5]) were undergraduate students of NCU. The young participants were paid 120 NTD (~4 USD)/h, whereas the elderly participants were given gifts (with a value equivalent to 120 NTD) for participation.

### Apparatus and Tasks

Participants were tested in a dim room without being interfered, where they were seated in an adjustable height chair next to a table. They performed an RT task (i.e., the MFT) and a discrete rapid-aiming task (i.e., Fitts' task). Based on the experience from the pilot study, the elderly participants generally feel more difficult to perform the Fitts' task and can take quite a long time to complete it. Therefore, the MFT always precedes the Fitts' task to allow participants to get familiar with the general experimental settings before being challenged with the more difficult task.

#### The MFT

In this study, in each trial, one, three, or five arrowheads were presented on some of the eight predefined locations on the invisible circular perimeter of a 3° radius. These arrowheads either point uniformly to the right or left or had a direction pointed to by the majority of them (e.g., two left and one right among three arrowheads; one right and four left among five arrowheads). The participant was instructed to determine the “majority direction” and press one of the two mouse buttons to indicate that direction, and the RT was defined as the duration between stimuli onset and the response. Participants practiced for a block of 16 trials before proceeding to the formal experimental trials. Fan et al. (2008) demonstrated that the RT increased as a linear function of the uncertainty that can be quantified by Shannon's entropy and was determined by the composition of the arrowhead directions in a “grouping search” manner. In other words, to solve a trial efficiently, one adopts a strategy to group and sample arrows with a majority size (i.e., more than half of the number of arrowheads in a trial) based on their directions. Accordingly, by defining the majority group size (1, 2, and 3 for set sizes of 1, 3, and 5, respectively) as the information unit and assuming that each sampled group is equivalent to one unit of information, the computational load, namely, the “level of uncertainty,” under the grouping search strategy can be quantified as log_*g*_(*s*), where *g* is the majority group size, i.e., a minimal number of arrows pointing in the same direction to be treated as the “majority,” and *s* is the total number of arrowheads to be scanned. To convert this quantity to *bits*, it is multiplied by log_2_(*g*), i.e., the computation load is log_2_(*g*)∙log_g_(*s*), which is equivalent to log_2_(*s*), where *s* is defined as the average number of arrowheads to be scanned in each condition. By manipulating the number of arrowheads and the composition of their binary pointing direction (3:0, 4:1, and 3:2), we adopted three different levels of computational load [1, 2.91, and 4.91 bits, respectively; see Fan et al. (2008) for how different compositions of pointing directions can be converted into the bit unit]. There were 54 trials for each load, which amounts to 162 trials in total.

#### The Fitts' Task

In each trial of the Fitts' task, participants moved the mouse pointer on the monitor from the starting location to a target disk as accurately and rapidly as possible. The MT in the Fitts' task was defined as the duration between the time points *t*_0_ and *t*_1_ after the target disk was presented on the screen, in which *t*_0_ indicates the time point when the cursor just moved outside the perimeter of the fixation disk (i.e., 10 pixels from its center), whereas *t*_1_ indicates the time point when the cursor just reached a position of which distance to the center of the target disk shorter than the target radius. The radius and center position of the target disk varied from trial to trial, which resulted in distinct ID, ID = log_2_(*A*/*D* + 1) (Fitts, 1954; later revised by MacKenzie, 1992), where *A* indicates the distance between the starting location and the center of the target and *D* indicates the target diameter. The different target disks appeared 5, 10, or 20 pixels in radius, and the distance between starting position and the target center ranged 160, 320, or 480 pixels. The ID here is conceptually equivalent to the load in MFT, and both are quantified in Shannon's entropy, *bit*. To consistently apply the nomenclature of the concept in MFT and Fitts' task, we hereby also call ID as load. Seven different combinations of *A* and *D* were adopted in this experiment, which resulted in seven distinct levels of load (2.32, 3.17, 3.7, 4.09, 4.64, 5.04, and 5.61 bits by taking binary logarithm on each level of load). Sixteen replications were presented for each load, except for 3.17 and 4.09 bits that had 32 trials. Participants experienced the balanced number of presentations of different combinations between distance and target size, totaled 144 trials presented in pseudorandom order. Two pairs of different combinations of target distance and diameters happened to render identical IDs, which doubled the number of trials. Before proceeding to the formal experimental trials, participants practiced a block of 16 trials to familiarize themselves with the task. To make the number of load levels comparable between Fitts' task and MFT, only the load levels of 2.32, 3.17, and 5.04 bits were taken into these analyses. It is noted that, in this study, the effective ID [ID_e_ = log_2_(*A*/*W*_e_ + 1), where *W*_e_ = 4.133 × SD; SD indicates the standard deviation of the distribution of the endpoint coordinates] was not computed because, given the definition of MT, the recorded end positions will always be inside the target disk. Therefore, the SD will never exceed the width of the target disk (*W*). The ID_e_ in this study, unlike other studies adopting velocity thresholds to determine the end position coordinates, is forced to be equivalent to the designed ID.

#### Data Analysis

The main dependent variable (DV) in the data analysis is the performance time, namely, RT in MFT and MT in Fitts' task. DV was trimmed adaptively *via* a principled approach that performs trimming in cycles: it first temporarily removes the slowest RT from the distribution and then calculates the mean of the sample. The cutoff value is calculated using a certain number of SD around the mean, with the value for SD being determined by the current sample size. In this procedure, required SD decreases with increased sample size (justification). The temporarily removed RT is then returned to the sample, and the fastest and slowest RTs are then compared with the cutoff and removed if they fall outside. This process is then repeated until no outliers remain, or until the sample size drops below four. The SD used for the cutoff is thus dynamically altered based on the sample size of each cycle of the procedure (Van Selst and Jolicoeur, 1994; Grange, 2015). All trimmed and error trials were excluded. Considering the representativeness of data included in the statistical analysis, participants with any condition that has fewer than 60% of trials remaining in a condition were excluded from further analysis. This resulted in the removal of 13 (9 elderly and 4 young) out of 84 participants from the subsequent analyses. The mean response accuracies of the remaining 71 participants are listed in Table 1.

**Table 1:** Accuracies and performance time (DV) of Fitts' task and MFT in each combination of age, task, and load.

**Age**	**Task**	**Load**	**Accuracy [CI 95%]**	DV [CI 95%]^*^
Old (*N* = 33)	Fitts	2.32	0.96 [0.94, 0.97]	1,268 [1,183, 1,353]
		3.17	0.93 [0.91, 0.94]	1,494 [1,408, 1,579]
		5.04	0.92 [0.90, 0.95]	2,240 [2,039, 2,440]
	MFT	1.00	0.96 [0.94, 0.98]	746 [621, 870]
		2.91	0.98 [0.97, 0.98]	1,365 [1,240, 1,490]
		4.91	0.79 [0.77, 0.82]	1,834 [1,712, 1,956]
You (*N* = 38)	Fitts	2.32	0.89 [0.86, 0.91]	570 [536, 603]
		3.17	0.88 [0.86, 0.91]	706 [673, 739]
		5.04	0.88 [0.85, 0.91]	1081 [1,037, 1,124]
	MFT	1.00	0.97 [0.95, 0.99]	537 [514, 559]
		2.91	0.99 [0.97, 1.00]	1040 [997, 1,083]
		4.91	0.90 [0.87, 0.93]	1476 [1,402, 1,549]

*Values in each cell indicate the mean performance time*

* *(DV indicates movement time in the Fitts' task and reaction time in MFT). Values in the square bracket indicate the upper and lower bounds of the 95% CI for each cell*.

The data were analyzed in the free statistical software environment R (version 3.6.3; R Core Team, 2021) using package *lme4* (version 1.1.23; Bates et al., 2015) for the model fitting procedure. The analysis was carried out with GLMMs on the trial level to optimally accommodate the non-Gaussian nature of the dependent measures and the continuous within-subject predictor (i.e., load) in the experimental design (Hox et al., 2017; Brauer and Curtin, 2018). Fixed-effects included task (i.e., MFT and Fitts), load (2.32, 3.17, and 5.04 bits for Fitts' task; 1, 2.91, and 4.91 bits for MFT), age group (i.e., elderly and young groups), and the interactions among the three factors. Additionally, age (in years), education (in years), and sex (male/female) was treated as nuisance variables where years of age and education were demeaned with respect to age group of each individual to amend the collinearity issue (formula = DV ~ 1 + age × task × load + age_year_ + education_year_ + sex). We coded task and age effects as +0.5/−0.5 contrasts (i.e., Fitts—MFT, elderly—young) to facilitate interpretations of results and centered load around mean of each participant (i.e., cluster-mean centering; Raudenbush and Bryk, 2002; Brauer and Curtin, 2018) to avoid the confounding between within-subject and between-subject associations (Enders and Tofighi, 2007).

Initially, we specified the random effects of the model in a “maximal manner” (Barr, 2013), where the intercept and all possible slopes are estimated in the random effect structure of the model. The subject was the random factor varying in mean DVs. We also assumed that subjects vary reliably in load and task effects (formula = ~1 + load × task|subject). The GLMM assumes that the mean DVs, load effects, and task effects of subjects distributed as inverse Gaussian functions around the respective fixed effects (i.e., the grand mean DV, the mean slope of load, and the mean difference between RT in MFT and MT in Fitts' task). This specification yields six variance/covariance component parameters for subjects. By comparing the Akaike's Information Criterion (AIC) fit statistics across GLMM with the identity, inverse, and log links, the inverse link turned out to offer the superior performance to the other links (see Supplementary Table 1, cf. Lo and Andrews, 2015).

This maximal model was subject to a stepwise procedure of model selection implemented in package *buildmer* (version 1.7.1). We set up the procedure to use the *maximal likelihood* and the *Nelder–Mead* optimizer to find the largest possible GLMM that still converges and then optionally performs stepwise elimination based on the change in log-likelihood as recommended by Matuschek et al. (2017). Summary statistics of the final model were also calculated based on Wald *z*-scores (Table 1).

Both fixed-effect and random-effect estimates of the GLMM are of theoretical interest in this study. As the population-level fixed-effect estimates inform about the effects of the independent variable over the whole group, the relationship among random-effect estimates provides a window for inspecting how different cognitive domains or processing mechanisms interact with each other. It is of theoretical interest to examine the strength of association between the estimates within and between tasks and compare the strength between different age groups: While stronger between-task association suggests more sharing of processing resources across cognitive domains, more vigorous within-task association indicate more interaction between general processing speed and processing efficiency specific to tasks.

## Results

The stepwise selection procedure settled on the maximal GLMM with all fixed effects and random effects remained. The total explanatory power of the model, which pitted both the fixed-effect and random-effect variance against the total variance, is substantial (conditional *R*^2^ = 0.948). Moreover, the part related to the fixed effects alone (marginal *R*^2^ = 0.907) is also very high. Thus, the rest of the “Results” section will describe the fixed- and random-effects and related statistics of the maximal model.

### Fixed Effect

**Figure 1:**
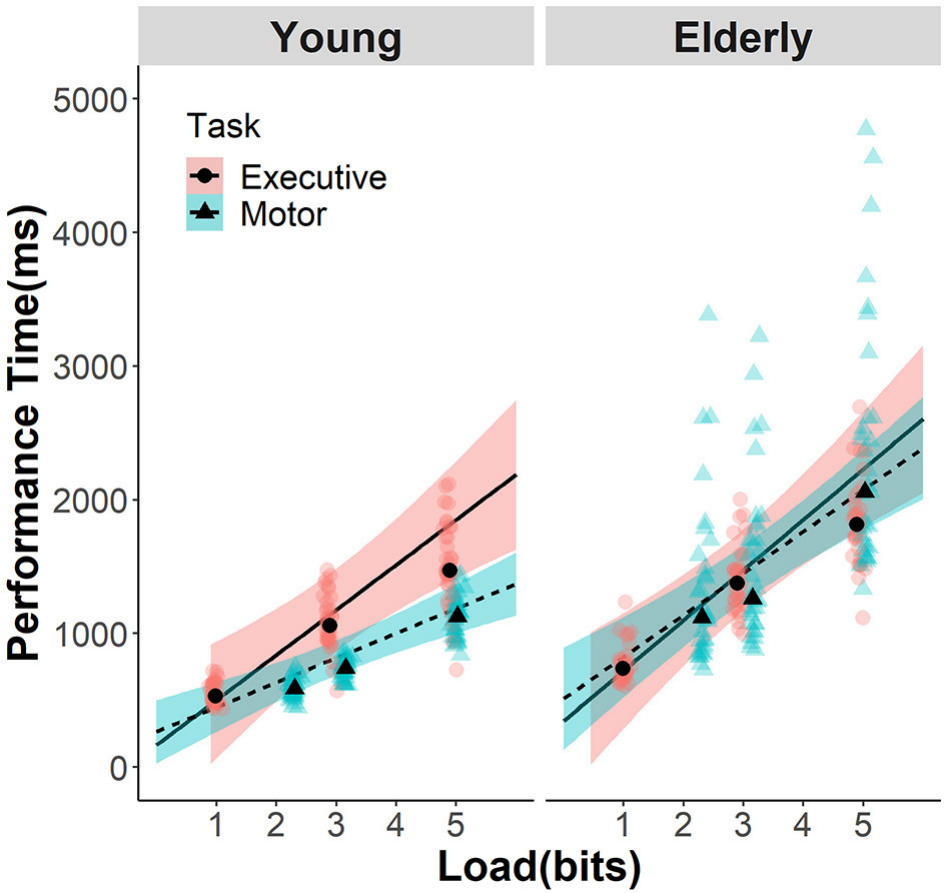
Efficiency functions for movement time in Fitts' task and reaction time in MFT. The data of young participants and GLMM estimates are presented in the left panel, while those for the elderly participants in the right panel. Data and estimates for MFT are indicated with dots, pink color, and solid lines, whereas those for Fitts' task were indicated with triangle, dashed lines, and cyan color. The shaded area indicates the 95% CI of the GLMM estimation. Transparent background symbols indicate the mean data of each individual participants, and the black foreground symbols indicate the mean of each group at the load level included in the analysis.

All fixed-effect estimates were significant (see Table 2). The highest order (three-way) interaction among age, load, and task was significant and is hereby dissected in detail with *post hoc z* tests implemented in the R package *emmeans*. Figure 1 clearly illustrates that linear functions between DV and load have distinct “slopes” among the combinations of age and task, which is equivalent to say at certain load level(s), performance times differ depending on the particular combination of age and task. Instead of going through each load level, we estimated the slopes of load that serve as linear contrasts at each level of the other two factors. This way, the two-way interaction of age and task on load slopes is effectively the three-way interaction among age, task, and load levels.

**Table 2:** Summary table of the final GLMM.

**Predictors**	**Inverse Gaussian/inverse link**			
	**Estimates**	**Standard error**	***CI***	**Statistic**
(Intercept)	−1.090^***^	0.010	−1.109~1.071	−110.551
Age	0.475^***^	0.016	0.442~0.507	28.855
Age:Load_cm	−0.108^***^	0.005	−0.119~−0.098	−19.933
Age:Task	0.370^***^	0.029	0.313~0.427	12.657
Age:Task:Load_cm	−0.053^***^	0.012	−0.076~−0.029	−4.299
Age_year.dm	0.002	0.001	−0.000~0.004	1.902
Education.dm	−0.002	0.002	−0.007~0.003	−0.817
F	Reference
M	0.015	0.012	−0.008~0.039	1.267
Load_cm	0.226^***^	0.003	0.220~0.231	83.098
Task	−0.141^***^	0.015	−0.170~−0.113	−9.678
Task:Load_cm	−0.004	0.006	−0.016~0.008	−0.599
**Random effects**				
σ^2^	0.01			
τ_00 subject_	0.00			
τ_11 subject.Task_	0.01			
τ_11 subject.Load_cm_	0.0			0
τ_11 subject.Task:Load_cm_	0.00			
ρ_01_	0.26			
	−0.95			
	−0.25			
ICC	0.44			
*N*_subject_	71			
Observations	14,844			
Marginal *R*^2^/conditional *R*^2^	0.907/0.948			
AIC	209348.393			
Log-likelihood	−104652.196			

*The fixed-effect estimates are listed in the upper part of the table, with the factor names indicated by the names in the rows. Age, age groups; Load_cm, load centered at the mean of each individual (weighted by the number of trials in each level); Task, MFT or Fitts' tasks; Age_year.dm, the actual age demeaned with respect to the mean age of each age group; Education_year.dm, the number of years in education demeaned with respect to the mean years of education within each age group; F or M, gender. Row names with factor names separated by colons indicate interaction effects. The middle part of the table lists the random effect parameters. Within-group (residual) variance: σ^2^; between-group-variance: τ_00_ (variation between individual intercepts and average intercept); random-slope-variance: τ_11_ (variation between individual slopes and average slope); random-intercept-slope-covariance: τ_01_; random-intercept-slope-correlation: ρ_01_*.

*** *p < 0.001; ICC, intra-class correlation. See Lüdecke (2021) for detailed explanations of the meaning of each symbol*.

With that, in the young group, MFT has a steeper slope (295 ms/bit, 95% CI = [281, 309]) than the Fitts' task (201 ms/bit, 95% CI = [190, 212]; *z* = 9.751, *p* < 0.001); whereas in the elderly group, the two tasks did not differ in slopes (MFT: 326 ms/bit, 95% CI = [309, 342]; Fitts: 352 ms/bit, 95% CI = [327, 377]; *z* = −1.680, *p* = 0.334). As for the comparison of the same task between age groups, both tasks showed significant differences (both *p* < 0.001; see Table 3). *Post-hoc* comparisons on the two-way interactions can be found in the Supplementary Information and will not be further explained here as they all depend on the significant higher-order interaction explored above.

**Table 3:** *Post-hoc* comparisons of the fixed-effect three-way interaction.

	**Young/MFT**	**Elderly/MFT**	**Young/Fitts**	**Elderly/Fitts**
Young/MFT	295			
Elderly/MFT	−31*	326		
Young/Fitts	94***	125***	201	
Elderly/Fitts	−57***	−26	−151***	352

**Figure 2:**
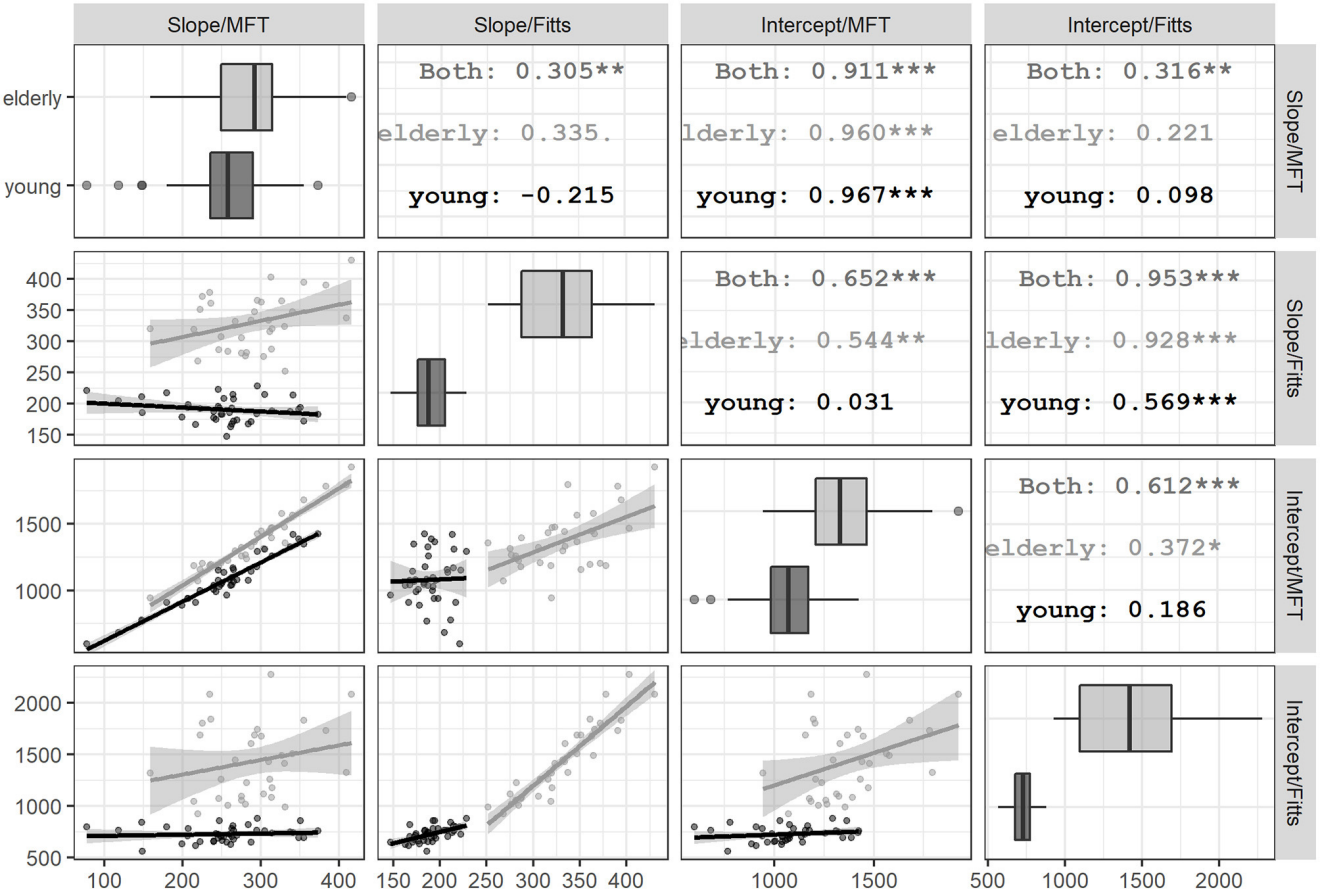
Pairwise correlations among parameters of efficiency functions within each age group. The scatter plots among the slopes and intercepts of MFT and Fitts' task within each age group are displayed in the off-diagonal cells. For the lower half of the matrix, the column and row titles indicate the parameters represented by the *X*- and *Y*-axes of each cell, respectively. For example, the scatter plot at the bottom left corner has column title “Slope/MFT” and row title “Intercept/Fitts,” which indicate dots in this cell represent the relationship between these two parameters in each task. In each cell, black line and dark dots indicate the young group, while gray line and light dots indicate the elderly group. The straight lines and shaded area indicate the simple linear regression lines between the *X*- and *Y*-parameters in a cell. Correlation coefficients in the upper-half cells indicate the correlation coefficients of the labeled group in the mirroring cell across the diagonal in the lower-half cells. The asterisks and dots suffixing the coefficients indicate their significance (*p* = 0.10, *p = 0.05, **p = 0.01, and ***p = 0.001). As there are 12 coefficients of interest (i.e., six pairwise combinations for the four parameters out of each age group), after correcting for family-wise error, only those with three asterisks should be considered significant. The diagonal cells show the boxplots of the young and elderly groups for the slopes and intercepts of each task.

*Row and column labels indicate < Age>/ < Task>. Diagonal cells: estimates of the load effect (i.e., slope) in the condition indicated by the row and column titles; lower triangle: estimates of differences between the conditions indicated by the row and column labels. Asterisks indicate the significance of the difference (same indication as those in Figure 2). For example, the cell with the row title “elderly/MFT” and column title “young/MFT” has the value of −31, indicating the “column – row” difference in slope between the two age groups for the MFT task [smaller for the column (young) group]*.

### Correlations Among Random-Effect Estimates

From the random-effect variance–covariance matrix of the GLMM (Table 2), it can be observed that the variance of all random-effect estimates (i.e., the three τ_11_ parameters) was quite substantial, suggesting unignorable individual differences in the way task and load modulate performance. Furthermore, there are sizable correlation coefficients between intercept (τ_00_) and slope of task and the interaction between task and load (τ_11_). Together these correlations justify closer inspection on how the random-effect parameters associate with one another in different age groups. The GLMM model did not estimate how age modulated the random effects of task and load because it only has two levels and thus not sufficient for fitting as a grouping random-effect. The marginal trend of the load was not computed by the *lme4* package and has to be estimated. Therefore, for each age group, we computed the marginal estimates of slopes and intercepts of the load predictor in both Fitts' task and MFT and then calculated the pairwise correlation coefficients among estimates (Figure 2). The elderly group showed a stronger pairwise correlation than the young group in all estimates, and almost all correlation coefficients reached significance (except for between the slopes of both tasks and between the Fitts' intercept and MFT slope). In contrast, for the young group, only the correlation between slopes and intercepts from the same task had a significant correlation. Moreover, the slope–intercept correlation in the Fitts' task is much weaker in the young group than the elderly group (*z* = 4.06, *p* < 0.001). Thus, the pattern of correlation among individual slopes and intercept within and between tasks suggests that elderly and young groups have a distinct relationship between different cognitive domains and processing mechanisms.

## Discussion

This study set out to examine how linear functions between processing time and computational load differ between cognitive domains and age groups. The results showed that at the group level, increment in the computational load resulted in statistically identical rates of MT and RT increment in the elderly group. In contrast, the young group showed a greater slope for RT of MFT than for MT of Fitts' law. Moreover, there are stronger associations among individual estimates of slopes and intercepts for the linear functions in the elderly group than the young group, both within the same task and between tasks.

With unified metrics for quantifying different cognitive functions and principled approaches in statistical analyses, the current findings added to the evidence of dedifferentiation of cognitive aging. It is plausible to assume that slopes of performance time as a function of processing loads indicate the information processing efficiency, and that correlation between slopes in different cognitive domains indicates the extent to how different cognitive domains share processing resources, adopting similar processing strategies, or modulated by some common internal factors (Sleimen-Malkoun et al., 2013). Thus, in the elderly people, the less distinction and the stronger correlation among the functional parameters of processing characteristics in the elderly group suggest a higher extent of commonality in processing resources/strategies between the domains of motor and executive functions.

In terms of potential mechanisms underlying the dedifferentiation, *central slowing* can be one of the candidates. For both MFT and Fitts' task, the elderly group has much steeper slopes and larger intercepts than the young group, indicating that the per-unit increase in processing load requires much more processing time and that even at the lowest possible processing load, the estimate of the processing time of elderly group is still much higher than the young group. It remains to be investigated that whether the longer processing time reflects genuine slowing or a more conservative strategy when facing cognitive challenges. Regardless of the cause, the slowing seems to mask the functional distinctions the cognitive system could have and lead to similar parameters in tasks measuring these functions. In contrast, the young cognitive system is not constrained by factors slowing down various processing streams and thus demonstrates higher efficiencies that preserve the idiosyncratic nature of different cognitive domains, as reflected in the distinctive slopes between tasks and weaker associations among functional parameters.

Hülür et al. (2015) suggested that the literature on the dedifferentiation hypothesis has reported mixed evidence, which seems to depend on research designs (longitudinal vs. cross-sectional) and nature of the sample (life span cohorts including young and middle-aged participants, or exclusively elderly). While both factors are plausible, in this study, we would like to suggest a third possibility, that is, the way in which different cognitive functions are measured and analyzed can also affect the outcomes and interpretations regarding the differential trends of aging. La Fleur et al. (2018) demonstrated how the inclusion of a test for speed of processing in the correlation with other abilities impacted the tendency of linear dedifferentiation among cognitive domains. As virtually all studies investigating the dedifferentiation hypothesis have relied on distinct ways of quantifying various cognitive domains, inevitably the analyses and interpretations were based on transformed scores. Taking the current results (and also Sleimen-Malkoun et al., 2013) together with previous reports, it is likely that a certain degree of variation in the inconsistencies among reports may be traced back to the distortion of measurements due to transformation and averaging of the performance index.

Although a previous study adopting a similar paradigm (Sleimen-Malkoun et al., 2013) also reached the same conclusion by contrasting the linear function between performance and load in Fitts' law and Hick–Hyman's law, this study extends the comparison across cognitive domains in two important aspects: first, the MFT prompts cognitive processing with an approach different from the multi-alternative S–R conflict resolution in the Hick–Hyman's law, providing convergent evidence to the dedifferentiation hypothesis. Second, in addition to demonstrating the difference between functions, with the aid of GLMM, and this study estimates individual variation in the linear functions and computes the correlations among them. The outcomes of stronger cross-domain and cross-mechanism correlations further support the dedifferentiation hypothesis. This research paradigm allowed more direct cross-domain comparisons than otherwise. Had different cognitive abilities been assessed with tasks that do not share the same scale in computational load, one would have to carry out the comparison based on summarized and transformed results, which may be statistically less powerful and only allows indirect comparison based on certain forms of ranking. This may constrain inferences that can be made with respect to life span trajectories in different cognitive domains.

A few caveats are worthy of note regarding this study: first, although this study aims at contrasting motor and executive functions, the performance in the Fitts' task may additionally involve visuomotor transformation as the participants actually controlled a computer mouse cursor to tap on the targets on the monitor. This experimental setting involved integrating visuospatial, proprioceptive, and motor information from the visual display and the hand holding the computer mouse, which posed a higher challenge than direct tapping. This may explain the much higher intercepts and slopes in this study. Second, the selection of three difficulty levels in processing load may not be optimal for estimating the functional relationship between processing loads and performance time. Although the comparison of the linear functions estimated from all loads higher than 3-bits in the Fitts' task from the current experiment led to similar outcomes and will not change the current conclusion (see Supplementary Material), future study may still consider more levels of loads for more robust estimation of the functional relationship. Third, in the Fitts' task, the MT in this study was determined based on geometrical (i.e., the distance to the center of the fixation disk or the target disk) rather than kinematic (e.g., velocity thresholds) landmarks. This makes the estimation of the movement amplitudes and endpoints of movements not entirely based on the ballistic part of the tapping movement and can involve the fine adjustment part in the final “homing-in” phase. Unfortunately, the mouse trajectories were not recorded during the experiment, and thus it is not possible to recover these more purely motor components of the measurements. The recorded endpoints may have underestimated spatial variations as they were confined within the target radius, and the potential inclusion of the homing-in phase may have inflated the MT.

It is a bit ironic to see the core information metric largely missing from experiments in cognitive psychology, a discipline claims to be founded on information processing. However, assessing all kinds of cognitive abilities under information-entropy-based manipulation may require a major overhaul of the tasks originally designed based on operational definitions that have been widely adopted. Fan (2014) proposed several cases of conceptualizing existing tasks for cognitive control under the information-theoretic framework. For example, the conflict effects originated from an interfering dimension, involving word-meaning in the color-word Stroop, flankers in the flanker task, or a global/local feature in a global/local selective attention task, can be treated as uncertainty difference between conflict and non-conflict conditions. In addition, one can also quantify the uncertainty with the surprise and entropy equations in paradigms investigating the oddball effect, Go/No-Go performance, or task switching, which all involve comparison between responses to high vs. low probability events. It remains a challenge to do likewise in other cognitive domains such as long-term memory and language processing.

## Conclusion and Applications

This study demonstrates an application of the information-theoretic approach in profiling motor and executive functions. Unlike numerous aging studies that compared different functions or established the relationship between functions *via* contrasting transformed scores or correlating divergent behavioral markers, the findings, in this study, provide supports for the dedifferentiation hypothesis of cognitive aging under the combinations of common efficiency metrics (i.e., bits) and principled statistical framework (i.e., GLMM) and show the group-level interaction among age, task, and computational load as well as the individual-level pattern of association among estimates of the relationship between computational load and performance time. This approach has both theoretical and practical values.

From the theoretical perspective, it may help to resolve inconsistencies in uncovering the changes in the structure of cognitive functions as one ages: as the same processing parameters (i.e., slopes and intercepts) were adopted to assess dedifferentiation of executive and motor domains, it avoids confounding stemming from biases in score transformation and standardization and allows to formulate more accurate assessment on the extent of dedifferentiation (see also Sleimen-Malkoun et al., 2013). This approach may also be applied to other domains that can be quantified with the same scheme. For example, the efficiencies of speech perception and production (e.g., Coupé et al., 2019) or sensory and perceptual processing (e.g., Plumbley and Abdallah, 2006) are both very well-quantifiable under the information-theoretic framework. By designing an appropriate experimental paradigm, the relationship among efficiency functions of various domains can be quantified and contrasted under unified information metrics and statistical models.

With respect to the practical perspective, depicting longitudinal trajectories across cognitive domains in a variety of developmental or clinical populations under this research framework may result in more accurate behavioral markers for identifying coherence between brain activations or rhythms underlying the interaction or dissociation between cognitive functions. The parameters of efficiency functions from different domains can form meaningful features for predicting the progression of cognitive aging or prognosis of neurological diseases.

## Data Availability Statement

The datasets presented in this study can be found in online repositories. The names of the repository/repositories and accession number(s) can be found below: OSFHOME: https://osf.io/h57pb/.

## Ethics Statement

The studies involving human participants were reviewed and approved by Research Ethics Committee of National Taiwan University. The patients/participants provided their written informed consent to participate in this study.

## Author Contributions

EC is the sole author who conceived and planned the study, coded the experimental tasks, performed the data analysis, and wrote the manuscript.

## Conflict of Interest

The author declares that the research was conducted in the absence of any commercial or financial relationships that could be construed as a potential conflict of interest.

## Publisher's Note

All claims expressed in this article are solely those of the authors and do not necessarily represent those of their affiliated organizations, or those of the publisher, the editors and the reviewers. Any product that may be evaluated in this article, or claim that may be made by its manufacturer, is not guaranteed or endorsed by the publisher.
