# Supplementary material for: Information-Theoretic Quantification of Dedifferentiation in the Aging of Motor and Executive Functions
Source: Front Aging Neurosci. 2021 Aug 20;13:634089. doi: 10.3389/fnagi.2021.634089 (PMC8418143; doi:10.3389/fnagi.2021.634089)
Supplement: Supplementary file 1 [file Data_Sheet_1.docx]

## Supplementary Information

The final GLMM and alternative models

Via the stepwise deletion procedure se selected the maximal model with Age, Task and Load as well as all of the interactions among them remained as fixed-effect factors, and Task, Load, and their interaction remained as random-effect factors in the model. The model selection procedure was also applied on models that assumed Gamma distribution for the response times. Moreover, for both Gamma and inverse Gaussian family, identity, log, and inverse links between the raw data and the model estimates were all examined. In other words, the combinations of two families of distribution (Gamma, inverse Gaussian) and three link functions (identity, log, and inverse) were all examined with the stepwise procedure of model selection. Among the final model of each of these six strains of selection, the inverse Gaussian family with the identity link has the lowest AIC/BIC values, and was explored in further details for its marginal effects and post hoc comparisons. Summaries of GLMM outcomes of all six final models are listed in supplementary Table S1.

Fixed effect outcomes of the final model

The final model's fixed-effect intercept, corresponding to Load = 0, Age = 0, and Task = 0, and subject at the population level, is at 1291.54 (SE = 12.57, 95% CI [1266.90, 1316.17], p < .001). As all participants

The effect of Load was significant (beta = 293.48, SE = 4.33, 95% CI [285.00, 301.96], p < .001). In general, one unit (bit) increase in loading of computation led to 293 ms increase of processing time. The effect of Age was significant (beta = 549.15, SE = 25.14, 95% CI [499.88, 598.42], p < .001). On average, the elderly were 549 ms slower than the young group. The effect of Task was significant (beta = -139.66, SE = 20.36, 95% CI [-179.57, -99.75], p < .001). Overall speaking, the movement time of Fitts task is 140 ms faster than the reaction time of MFT. All of the two-way and the three-way interactions were significant, including Task 🞨 Age (beta = 532.02, SE = 40.73, 95% CI [452.20, 611.85], p < .001), Age 🞨 Load (beta = 90.88, SE = 8.66, 95% CI [73.91, 107.85], p < .001), Task 🞨 Load (beta = -33.84, SE = 9.18, 95% CI [-51.84, -15.85], p < .001). Outcomes of post-hoc comparisons on the two-way interactions are listed in Table S2.

Table S1. Summary of GLMM models adopting inverse and log links

|  | **identity** | | | | **inverse.gaussian inverse** | | | | **log** | | | |
| --- | --- | --- | --- | --- | --- | --- | --- | --- | --- | --- | --- | --- |
| *Predictors* | *Estimates* | *std. Error* | *CI* | *Statistic* | *Estimates* | *std. Error* | *CI* | *Statistic* | *Estimates* | *std. Error* | *CI* | *Statistic* |
| (Intercept) | 1343.78 ^***^ | 13.33 | 1317.65 ~ 1369.91 | 100.79 | -1.09 ^***^ | 0.01 | -1.10 ~ -1.07 | -137.14 | 7.01 ^***^ | 0.01 | 6.99 ~ 7.03 | 757.52 |
| Age | 711.91 ^***^ | 26.67 | 659.65 ~ 764.18 | 26.70 | 0.46 ^***^ | 0.02 | 0.42 ~ 0.49 | 28.71 | 0.34 ^***^ | 0.02 | 0.31 ~ 0.37 | 22.17 |
| Age:Task | 796.62 ^***^ | 45.52 | 707.41 ~ 885.84 | 17.50 | 0.38 ^***^ | 0.03 | 0.32 ~ 0.43 | 12.88 |  |  |  |  |
| Age_year.dm |  |  |  |  | 0.00 | 0.00 | -0.00 ~ 0.00 | 0.74 |  |  |  |  |
| Education.dm |  |  |  |  | -0.00 | 0.00 | -0.00 ~ 0.00 | -0.01 |  |  |  |  |
| Load_cm | 254.05 ^***^ | 3.19 | 247.79 ~ 260.30 | 79.61 | 0.23 ^***^ | 0.00 | 0.22 ~ 0.23 | 86.19 | 0.25 ^***^ | 0.00 | 0.25 ~ 0.26 | 83.71 |
| Load_cm:Age | 71.67 ^***^ | 6.38 | 59.16 ~ 84.18 | 11.23 | -0.11 ^***^ | 0.01 | -0.12 ~ -0.10 | -20.36 |  |  |  |  |
| Load_cm:Age:Task | 71.43 ^***^ | 12.76 | 46.41 ~ 96.44 | 5.60 | -0.05 ^***^ | 0.01 | -0.08 ~ -0.03 | -4.14 |  |  |  |  |
| Load_cm:Task | -26.47 ^***^ | 6.38 | -38.98 ~ -13.97 | -4.15 | 0.00 | 0.01 | -0.01 ~ 0.02 | 0.52 |  |  |  |  |
| Task | 120.21 ^***^ | 22.76 | 75.61 ~ 164.82 | 5.28 | -0.14 ^***^ | 0.01 | -0.17 ~ -0.12 | -9.91 | -0.06 ^**^ | 0.02 | -0.09 ~ -0.02 | -3.19 |
| **Random Effects** | | | | | | | | | | | | |
| σ^2^ | 0.00 | | | | 0.00 | | | | 0.00 | | | |
| τ_00_ | 11603.95 _subject_ | | | | 0.00 _subject_ | | | | 0.01 _subject_ | | | |
| τ_11_ | 32799.21 _subject.Task_ | | | | 0.00 _subject.Load_cm_ | | | | 0.02 _subject.Task_ | | | |
|  |  | | | | 0.01 _subject.Task_ | | | | 0.00 _subject.Load_cm_ | | | |
|  |  | | | | 0.00 _subject.Load_cm:Task_ | | | |  | | | |
| ρ_01_ | 0.84 _subject_ | | | | -0.92 | | | | 0.53 | | | |
|  |  | | | | 0.26 | | | | -0.02 | | | |
|  |  | | | | -0.43 | | | |  | | | |
| ICC | 1.00 | | | | 0.99 | | | | 0.99 | | | |
| N | 71 _subject_ | | | | 71 _subject_ | | | | 71 _subject_ | | | |
| Observations | 14844 | | | | 14844 | | | | 14844 | | | |
| Marginal R^2^ / Conditional R^2^ | 0.955 / 1.000 | | | | 0.955 / 0.999 | | | | 0.947 / 1.000 | | | |
| AIC | 207513.091 | | | | 209344.622 | | | | 207264.213 | | | |
| log-Likelihood | -103744.545 | | | | -104651.311 | | | | -103621.106 | | | |
| ** p<0.05   ** p<0.01   *** p<0.001* | | | | | | | | | | | | |

Note. Within-group (residual) variance: **σ^2^**; between-group-variance: **τ_00_** (variation between individual intercepts and average intercept); random-slope-variance: **τ_11_** (variation between individual slopes and average slope); random-intercept-slope-covariance: τ_01_; random-intercept-slope-correlation: **ρ_01_**_._  * p<0.05 ** p<0.01 *** p<0.001

Table S2. Post hoc comparisons of two-way interactions

| **Age 🞨 Task** | | | | |
| --- | --- | --- | --- | --- |
|  | *young/MFT* | *elderly/MFT* | *young/Fitts* | *elderly/Fitts* |
| *young/MFT* | 1220 |  |  |  |
| *elderly/MFT* | -283*** | 1503 |  |  |
| *young/Fitts* | 406*** | 689*** | 814 |  |
| *elderly/Fitts* | -409*** | -126*** | -815*** | 1629 |
| **Age 🞨 Load** | | | | |
|  | young | elderly |  |  |
| young | 248 |  |  |  |
| elderly | -91*** | 339 |  |  |
|  |  |  |  |  |
| **Task 🞨 Load** | | | | |
|  | MFT | Fitts |  |  |
| MFT | 310 |  |  |  |
| Fitts | 34** | 277 |  |  |

Note. For each grids of each interaction, the diagonal cells indicate the values for each condition, the lower triangles indicate the difference between the condition appear “earlier” in the table (more left columns or upper rows), and the upper triangles indicate the *p* values of the comparison (adjusted with Tukey method when there are more multiple pairs of values). In the Age **🞨** Load and Task **🞨** Load interaction, the diagonal values indicate the estimated slope of Load in the different levels of the other factor.

Table S3. GLMM outcomes with all Loads above 3-bits in the Fitts’ task

This table extend the number of the Load levels to use more information collected in the experiment while avoiding the load level controversial in the Fitts’ law.


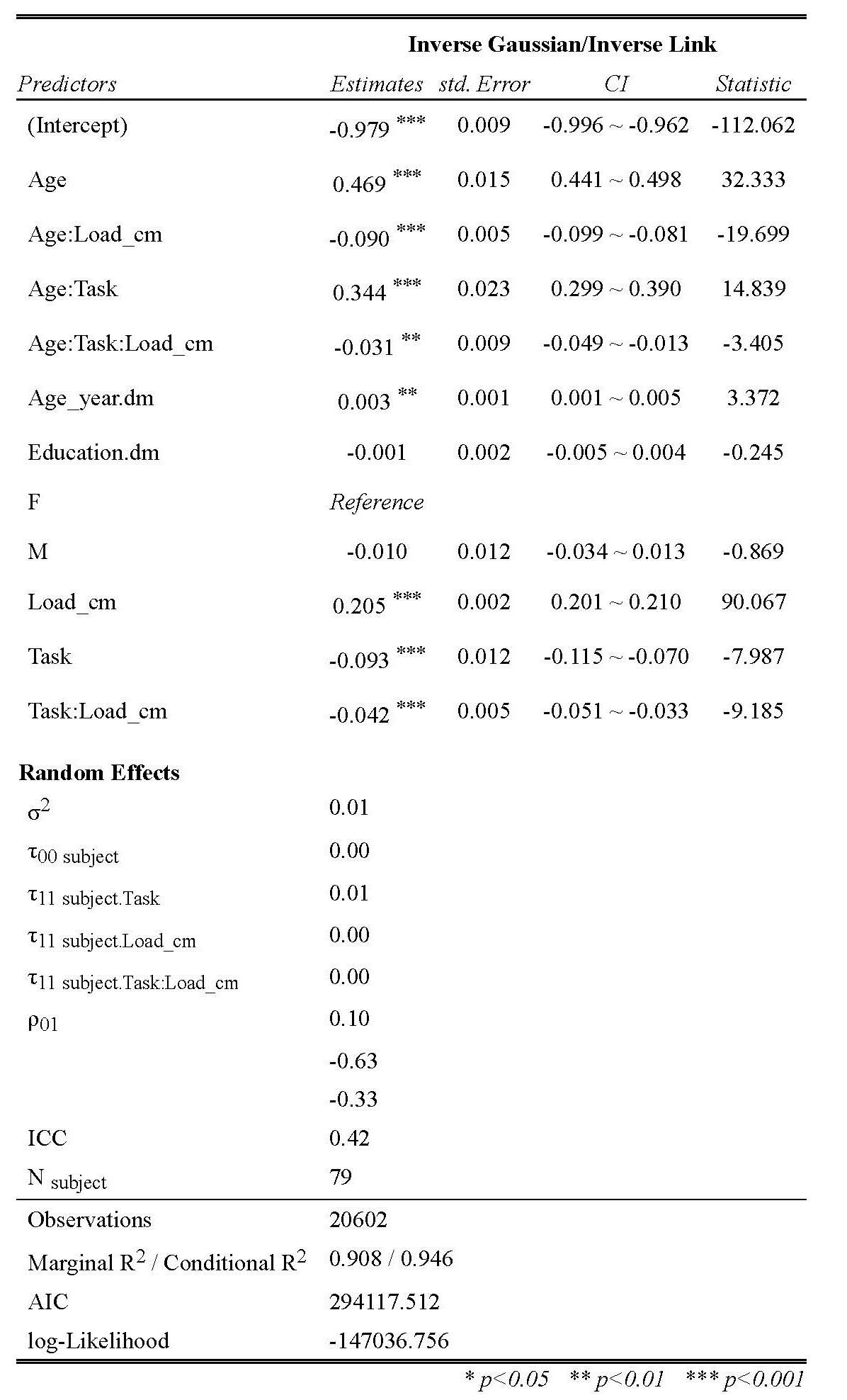


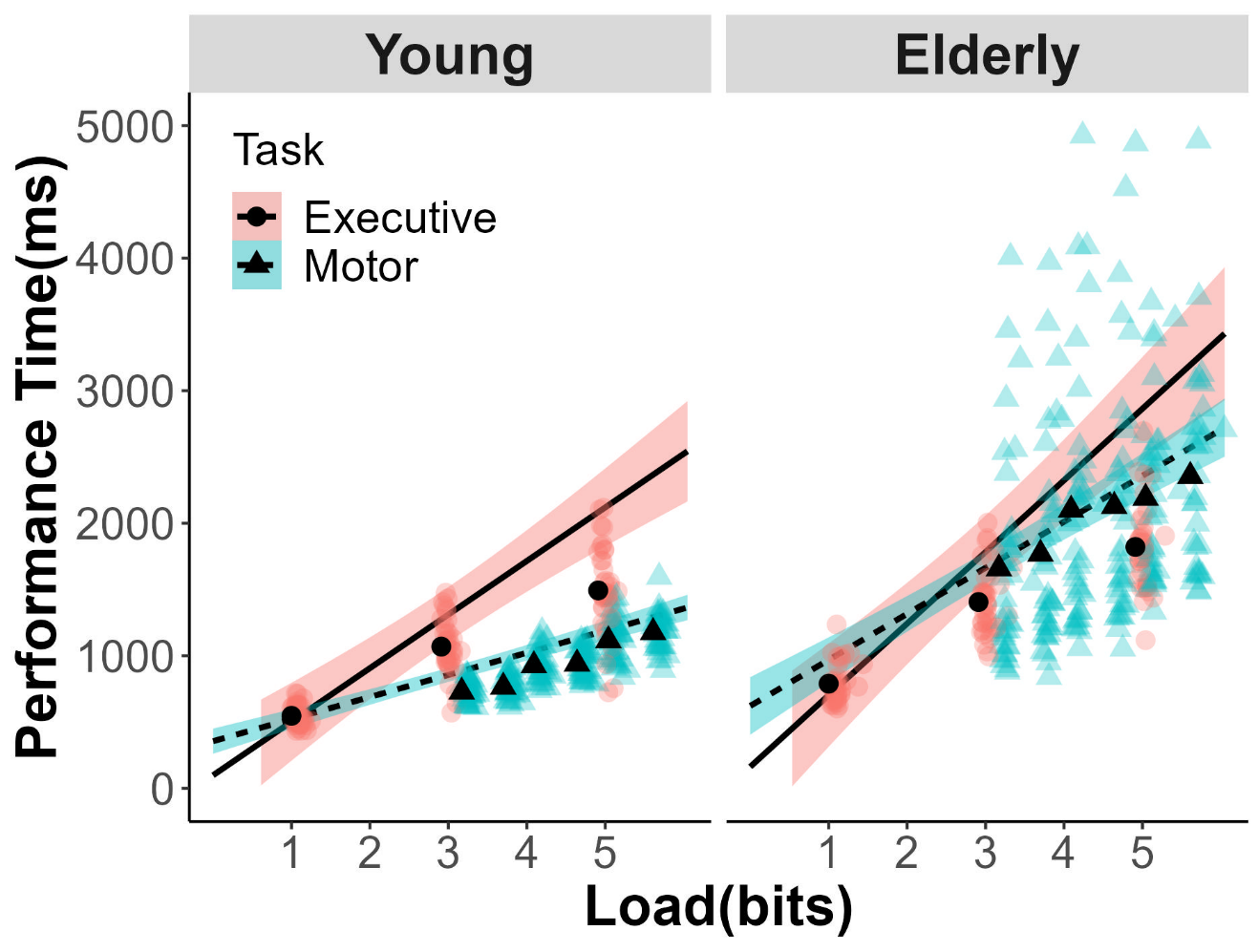


**Figure S1** | **Efficiency functions for movement time in Fitts’ task and reaction time in MFT (with all Fitts’ load level > 3 bits)**
